# Foundations of Gerophysics

**DOI:** 10.18632/aging.206378

**Published:** 2026-05-14

**Authors:** Maximilian Unfried, Weihan Huai, Kamil Pabis, Sandra Jose, Zhi Meng Lim, Uri Alon, Marija Cvijovic, Nir Eynon, Peter Fedichev, Yumi Kim, Leong Kim Whye, Csaba Kerepesi, Woon-Puay Koh, Dmitrii Kriukov, Matt Kaeberlein, Feng Ling, Glen Pridham, Michael Rera, Steffen Rulands, Andrew Rutenberg, Kumar Selvarajoo, Ben Shenhar, Morten Scheibye-Knudsen, Andrei E. Tarkhov, Andrew Teschendorff, Weilan Wang, Ee Hou Yong, Yifan Yang, Jan Gruber, Brian K. Kennedy

**Affiliations:** 1 Healthy Longevity Translational Research Program, Yong Loo Lin School of Medicine, National University of Singapore 117456, Singapore; 2 Department of Biochemistry, Yong Loo Lin School of Medicine, National University of Singapore 117596, Singapore; 3 Department of Molecular Cell Biology, Weizmann Institute of Science, Rehovot 76100, Israel; 4 Department of Mathematical Sciences, Chalmers University of Technology, Gothenburg 412 96, Sweden; 5 Department of Mathematical Sciences, University of Gothenburg, Göteborg 412 58, Sweden; 6 Australian Regenerative Medicine Institute (ARMI), Faculty of Medicine, Nursing and Health Sciences, Monash University, Melbourne, Victoria 3800, Australia; 7 GERO PTE. LTD., Singapore 069535, Singapore; 8 ieumBio Co. Ltd., and Biomedical Engineering, UNIST, Ulsan 44919, Republic of Korea; 9 Mechanobiology Institute, National University of Singapore, Singapore 117411, Singapore; 10 Institute for Computer Science and Control (SZTAKI), Hungarian Research Network (HUNREN), Budapest 1111, Hungary; 11 HUN-REN-SZTAKI-SU Rejuvenation Research Group, Office for Supported Research Groups (TKI), Hungarian Research Network (HUN-REN), Budapest 1052, Hungary; 12 Institute for Human Development and Potential (IHDP), Agency for Science, Technology and Research (A*STAR), Singapore 117609, Singapore; 13 Artificial Intelligence Research Institute, Moscow 121170, Russian Federation; 14 Skolkovo Institute of Science and Technology, Moscow 121205, Russian Federation; 15 Optispan, Tukwila, WA 98168, USA; 16 Institute of High Performance Computing (IHPC), Agency for Science, Technology and Research (A*STAR), Singapore 138632, Singapore; 17 Department of Physics, National University of Singapore, Singapore 117551, Singapore; 18 Université Paris Cité, Functional and Adaptive Biology, UMR 8251, CNRS, F-75013 Paris, France; 19 Ludwig-Maximilians-Universität München, Arnold-Sommerfeld-Center for Theoretical Physics, München 80333, Germany; 20 Department of Physics and Atmospheric Science, Dalhousie University, Halifax B3H 4R2, Nova Scotia, Canada; 21 Bioinformatics Institute (BII), Agency for Science, Technology and Research (A*STAR), Singapore 138632, Singapore; 22 Synthetic Biology Translational Research Program, Yong Loo Lin School of Medicine, National University of Singapore (NUS), Singapore 117456, Singapore; 23 School of Biological Sciences, Nanyang Technological University, Singapore 637551, Singapore; 24 Center for Healthy Aging, Department of Cellular and Molecular Medicine, University of Copenhagen, Copenhagen 2200, Denmark; 25 Retro Biosciences Inc., Redwood, CA 94063, USA; 26 Shanghai Institute for Nutrition and Health, Chinese Academy of Sciences, Shanghai 200031, China; 27 NUS Academy for Healthy Longevity, Yong Loo Lin School of Medicine, National University of Singapore 119228, Singapore; 28 Division of Physics and Applied Physics, School of Physical and Mathematical Sciences, Nanyang Technological University, Singapore 637371, Singapore; 29 Center for Interdisciplinary Studies, Westlake University, Hangzhou, Zhejiang, China; 30 Department of Physiology, Yong Loo Lin School of Medicine, National University of Singapore 117593, Singapore

**Keywords:** gerophysics, geroscience, aging biology, longevity, complex systems, theoretical physics

## Abstract

The inaugural Global Conference on Gerophysics convened 160 researchers across physics, biology, computation, and medicine in Singapore on March 5–6, 2025. With 31 speakers, the two-day event explored how physical laws, and quantitative principles unify aging science, linking biological processes to longevity patterns. Sessions covered diverse aging aspects, from developmental stages to species comparisons. The meeting showcased innovative methods to study aging, emphasizing data-driven insights. A central theme was bridging theory and experiment to advance understanding. It concluded with a consensus around (1) shared multi-modal datasets; (2) physics-based definitions for “aging”, “rejuvenation”, and “healthspan”; (3) models predicting intervention outcomes; and (4) translational links between non-human species and human research. These priorities outline a practical path for a quantitative, predictive Gerophysics, building on the 2025 conference’s insights to shape future aging research.

## INTRODUCTION

Aging unfolds across molecules, cells, tissues, and populations; yet it shows recurring regularities. Gerophysics (Box 1) is the effort to capture those regularities with physical laws, identifying the key state variables of aging, the constraints and invariants that shape their trajectories, and the forces that drive change. It asks which quantities move together, where tipping points and trade-offs arise, and how interventions can steer trajectories towards a longer healthy life. The inaugural Global Conference on Gerophysics hosted in Singapore on March 5–6, 2025 by the Healthy Longevity Translational Research Programme, Yong Loo Lin School of Medicine, National University of Singapore brought together diverse scholars across physics, biology, computation, and medicine to exchange ideas and lay the groundwork for a predictive, falsifiable science of aging (Figure 1).

**Figure 1:**
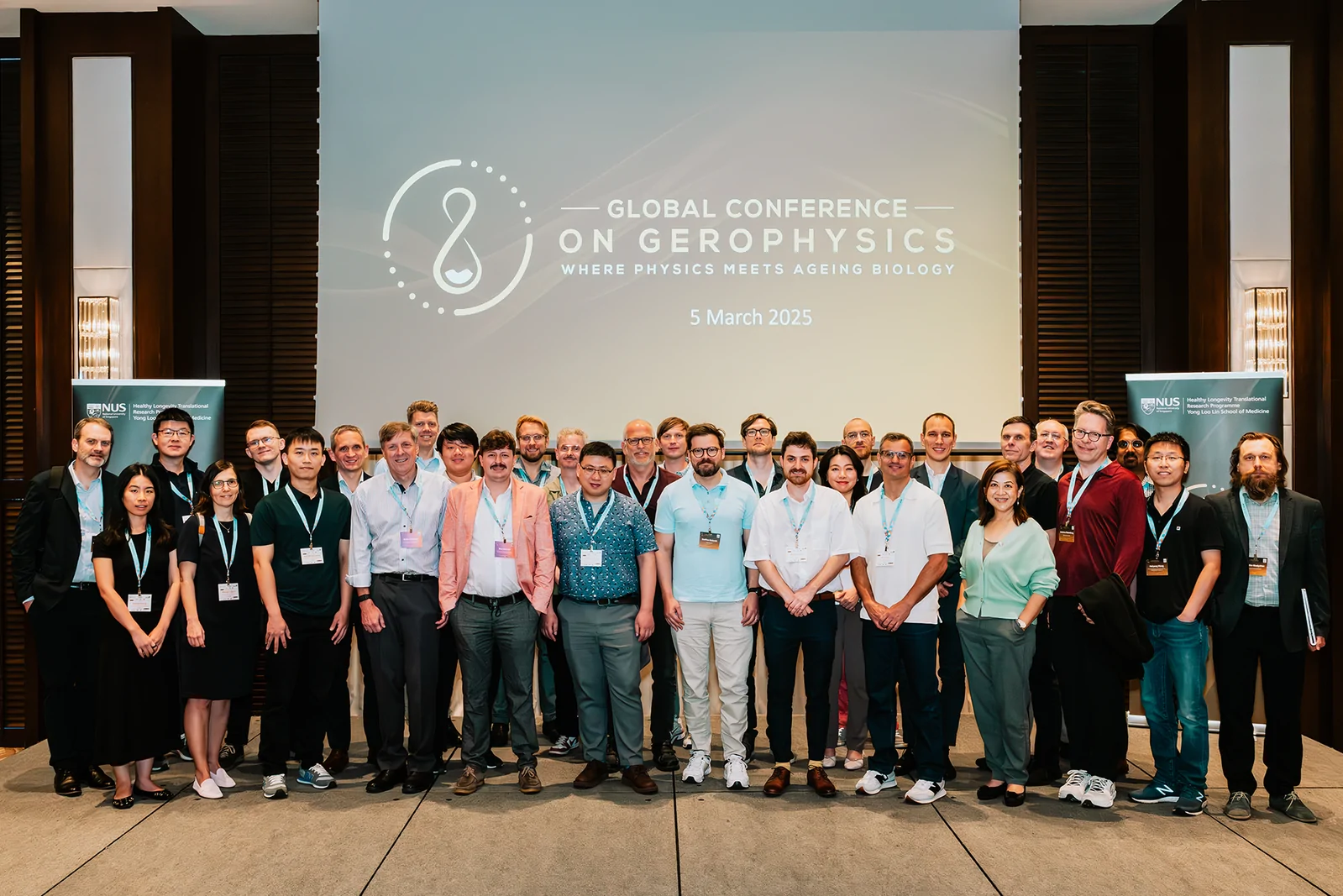
Speakers and chairs of the conference from left to right: Sebastien Thuault, Weilan Wang, Ee Hou Yong, Marija Cvijovic, Dmitrii Kriukov, Leong Kim Whye, Peter Fedichev, Brian K. Kennedy, Morten Scheibye-Knudsen, Weihan Huai, Maximilian Unfried, Jan Gruber, Peter James Mullen, Yifan Yang, Uri Alon, Andrei E. Tarkhov, Steffen Rulands, Glen Pridham, Ben Shenhar, Yumi Kim, Michael Rera, Nir Eynon, Csaba Kerepesi, Woon-Puay Koh, Matt Kaeberlein, Andrew Teschendorff, Andrew Rutenberg, Kumar Selvarajoo, Haiyang Wang, Vadim Gladyshev. Missing in the picture are Feng Ling and Kamil Pabis.

Box 1 **What is Gerophysics?**

Gerophysics – the Physics of Aging - is an emerging interdisciplinary field that applies principles from theoretical physics such as non-equilibrium thermodynamics, dynamical systems theory, network science, and stochastic processes, to decode the fundamental mechanisms of biological aging. It represents a paradigm shift in aging research by treating aging not just as a biological process but as a physical one governed by universal laws, like entropy production, phase transitions, and energy landscapes of damage accumulation. This perspective emphasizes the stability of high-dimensional physiological states and supports quantitative, falsifiable models with observable critical transitions that link molecular damage, repair, and information flow to organism-level trajectories such as frailty, loss of resilience, and Gompertzian mortality. The goal is to build predictive models that bridge changes across biological scales: from subcellular to organism level, and timescales from cellular turnover to lifespan. Ultimately, gerophysics aims to provide a unifying mathematical scaffold that turns aging research from descriptive catalogs into a predictive discipline, that can inform theory-driven biological experimentation and enable the discovery of novel interventions to extend healthy lifespan.

This meeting report distils the main ideas, points of convergence and open questions that emerged. We organize the content into thematic highlights, followed by key takeaways (Box 2) and a brief outlook that proposes shared datasets and open benchmarks, physics-grounded definitions, standards for uncertainty, and prioritized model-to-experiment workflows. The goal is not a comprehensive review, but a practical snapshot of where Gerophysics stands and what is needed next to build a predictive, testable and ultimately translational science of aging.

Box 2 **Key takeaways**

1. Iterative Model-Experiment Loop: Physics models guide experiments; data refines models for Gerophysics discovery.
2. Causal, Testable Models: Predict responses to interventions with benchmarks for validation.
3. Physics-Grounded Definitions: Standardize “aging”, “rejuvenation,” and “healthspan” with state variables.
4. Shared Datasets: Promote multi-modal datasets and open benchmarks for reproducibility.
5. Interdisciplinary Training: Bridge biology, physics, and computation for synergistic progress.

## Thematic highlights

### The emergence of Gerophysics: integrating physical laws into aging science

The first session focused on applying physics laws to understand the aging process. In the first talk, Uri Alon from the Weizmann Institute of Science presented his group’s work on how to model three aging patterns - exponentially rising death with late slowdown, exponentially rising disease incidence with late decline, and linear decline of physiological function with age. He assumed that there is a driver of aging that is produced and removed, and he used p16 luciferase on mice and starving *E. coli* to model this process. He presented a differential equation - the saturating removal (SR) model - that has damage production that rises with time (due to accumulating damage producing units) and damage removal process that saturates at high damage plus noise [1]. This equation can explain all three aging patterns. Furthermore, the model can analyze the shape of the survival curve to tell if geroprotective interventions act on damage production (survival scaling) or damage removal/threshold (survival steepening). This allowed identification of drivers of aging from large scale yeast gene deletion studies. In this way the model identifies two modes of aging, one driven by chromosome changes and double stranded breaks and the other by lysosome/mitochondrial changes.

Dr. Yifan Yang, an assistant professor in Westlake University, further expanded the SR model proposed by Uri Alon to show how this model can help people identify interventions that can suppress the sickspan [2]. He showed that based on the SR model, longevity interventions act by reducing the damage production rate and/or increasing the damage removal rate, both of which are inherently age-dependent processes in this model. He theorized that interventions that prevent damage production will lead to scaling of both lifespan and sickspan, which leads to scaling of the lifespan curves, while interventions that increase damage removal rate can compress the sickspan, leading to the steepening of the survival curve. Supporting this theory, Dr. Yang further showed that steepening the survival curve can compress sickspan in multiple species ranging from invertebrates to mice, supporting his theory. Next, he applied his theory to mice and looked for interventions that can steepen the lifespan curve using the lifespan curves from the Interventions Testing Program (ITP) run by the National Institute of Aging (NIA). He identified 4 classes of interventions that can steepen the survival curve, focusing on vasculature protection. When expanding the analysis to 26 more interventions reported from wider literatures, he identified two more classes (improvement of VEGF and senolytics) that could steepen the curve, suggesting that these classes of intervention can be potent candidates for delaying sickspan.

Professor Marija Cvijovic (University of Gothenburg) opened her talk by highlighting how mathematical models can “find simplicity in complexity” when studying the highly intricate process of aging. By integrating diverse data and enabling *in silico* experiments, such models offer powerful tools to understand aging mechanisms. She presented her group’s work on developing new computational tools, such as a Bayesian state-space inference framework [3] and a fast parameter-estimation Julia package [4] to calibrate mechanistic models efficiently. She introduced the large -multi-scale aging model [5], which integrates nutrient signalling, metabolism, damage accumulation, and growth, enabling exploration of metabolic changes as the cell ages and becomes exposed to stress and protein damage. As the cell gets older, the timing and pattern of normal metabolic states change, so-called metabolic phases. By accounting for these altered patterns, the model can help guide metabolic interventions for lifespan control, by identifying points where interventions might have different effects depending on when they occur in the cell’s life. This was exemplified by certain enzymes that shorten lifespan when perturbed in a specific metabolic phase, but do not affect or even prolong lifespan when perturbed over the whole life. She concluded by advocating for combining machine learning and mechanistic modelling into uniform data-driven mechanistic frameworks to pave the way for personalized metabolism-focused strategies to modulate aging.

## Fundamental principles: physics derived models for understanding aging

Bringing physics into dialogue with biology, this session showcased how aging can be captured through mathematical and thermodynamic models. From phase-based transitions in organismal decline to stability–instability regimes in gene networks, speakers demonstrated how physical laws can reveal unifying principles of lifespan and mortality.

Dr. Michael Rera, a CNRS researcher at the Department of Functional and Adaptive Biology, Université Paris Cité, presented a two-phase model of aging based on intestinal barrier dysfunction. Using *Drosophila melanogaster* as a model, he developed a simple assay in which flies are fed food containing a blue dye to assess intestinal permeability [6].

Flies with a leaky gut absorb the dye, which spreads throughout their body, giving them a characteristic blue coloration, hence the name “Smurf” phenotype.

Dr. Rera showed that flies consistently transition to the Smurf state shortly before death, and that the proportion of Smurf flies increases over time, indicating that intestinal permeability is an age-dependent physiological marker. Interestingly, once flies become Smurfs, their remaining lifespan is relatively constant and independent of their chronological age at the time of transition.

These observations led Dr. Rera to propose a discontinuous, two-phase model of aging, consisting of a first phase from youth to the onset of Smurfness and a second phase from Smurfness to death [7]. Importantly, this two-phase aging pattern appears to be conserved across species, having been observed not only in fruit flies but also in nematodes, zebrafish [8] and mice [9].

Dr. Peter Fedichev, the founder of the preclinical-stage biotechnology company Gero, and Prof. Jan Gruber, an associate professor at the National University of Singapore, presented their phenomenological theory of aging, recently synthesised in a joint review [10]. Their theory is based on considering aging as damage driven stability-instability phase transition in gene regulatory networks, where most species operate close to the stable-unstable boundary. Short-lived species such as flies, worms, and mice exemplify the unstable regime, showing exponential dynamics in biomarkers trajectories and mortality acceleration. In contrast, humans mostly occupy the stable regime**,** where resilience declines gradually while entropy-driven configuration changes accumulate irreversibly. Within this framework, the authors introduce a thermodynamic quantity they term the “effective temperature”, a key parameter that quantifies the intensity of stochastic fluctuations in the network and controls the difference between average and maximum lifespan, making it a prime target for interventions in humans. This theory explains why there is no variable change exponentially during aging in humans: humans are stable animals and our resilience declines during aging. Dr. Fedichev further showed that entropy is the main driving factor of loss of resilience, and the way to reduce entropy accumulation rate is to target the maximum lifespan of humans. Hence, by tying aspects of aging to the Second Law of Thermodynamics, the theory predicts that rejuvenation can only be achieved on an unstable animal.

Prof. Gruber presented an application of the new phenomenological theory of aging to model organisms, showing how this synthesis of theory and experiment reconciles puzzling experimental findings in *C. elegans*. In *C. elegans*, the burden of mitochondrial DNA (mtDNA) lesions increases with age. Yet even when mtDNA damage was further increased experimentally by UV or γ-irradiation, lifespan was unchanged, challenging simple damage-accumulation models in which mtDNA lesions are a primary determinant of longevity [11]. The framework, which treats short-lived organisms as unstable dynamical systems where stochastic noise scales intrinsic instability without requiring accumulated damage as a driver of aging, explains not only these paradoxical results but also the unexpectedly large benefits of late-life interventions in key aging pathways in these species. Using numerical simulations, Prof. Gruber demonstrated how this approach can, for example, account for the striking extension of lifespan when *daf-2* is depleted very late in life. This work connects universal survival patterns with measurable biomarker dynamics in a manner consistent with principles from dynamical systems and statistical physics. Together, it positions phenomenological physics as a central pillar of Gerophysics.

Mr. Ben Shenhar, a PhD student in the Uri Alon Lab, presented his work on reassessing the heritability of human lifespan. The widely cited estimates of lifespan heritability, based on Scandinavian twin studies, report low heritability of roughly 25% [12]. Shenhar demonstrated that in these historical cohorts, extrinsic mortality, defined as deaths which are caused by factors that originate outside the body, such as by infections or violence, was roughly ten times higher than it is today, making it a strong confounder. Elevated extrinsic mortality reduces median lifespan differences between genetic groups while inflating variation within groups, both of which diminish twin correlations and lead to underestimates of heritability. To address this, Shenhar developed a method to isolate lifespan heritability from intrinsic mortality alone. He introduced heterogeneity into two mortality models - the saturated removal model [1] and the Makeham-Gamma-Gompertz model - enabling simulation of genetic groups and thereby separation of intrinsic from extrinsic effects. Using this approach, he found that intrinsic heritability of lifespan rises to about 50%, suggesting a substantial genetic contribution once extrinsic mortality is accounted for [13].

## Synergizing physics and AI: computational approaches to aging biology

This session explored how computational methods—ranging from systems biology and large-scale data mining to AI-driven molecular design—are reshaping the study of aging mechanisms and accelerating the search for geroprotective interventions.

Dr. Kumar Selvarajoo, Senior Principal Investigator at the Bioinformatics Institute, A*STAR, discussed how systems biology approaches can be used to understand and target the complex mechanisms underlying aging and age-related diseases. He emphasized that many drugs with potential geroprotective effects were originally developed to treat conditions such as diabetes, infections, or immune disorders rather than to modulate aging itself. This overlap highlights that aging is a multifactorial process sharing common molecular and systemic pathways with these diseases, suggesting that insights from existing therapeutics may help uncover new strategies for promoting healthy aging. He presented his previous works on how system biology approaches have been applied to deconvolute complex diseases and to achieve certain goals in the complex system, such as enhancing apoptosis in cancer [14], understanding time-dependent cellular state transition [15], and personalized breast cancer drug responses through combination of multi-omics and machine learning [16]. Dr. Selvarajoo suggests that a multi-dimensional, systems-level understanding at the multi-omics scale can reveal new opportunities to repurpose existing therapeutics and develop innovative strategies for promoting healthy aging, ultimately advancing personalized aging medicine through digital twin approaches [17].

Prof. Matt Kaeberlein, the Chief Executive Officer at Optispan Inc., and affiliate Professor of Oral Health Sciences at the University of Washington, presented the application of AI to screen for potential anti-aging compounds. In a provocative talk, he compared the current interventions on mice and showed that none of them so far outcompete calorie restriction, suggesting that there is still a long way to go for solving aging. He then presented the million-molecule challenge, which is powered by WormBot-AI to screen over 1 million molecules on *C. elegans* for their effect on worms lifespan [18]. WormBot tracks phenotypes of worms to analyze their lifespans and Prof. Kaeberlein showed that there exist interesting patterns of classes and pathways when looking into the molecular targets of drugs that showed lifespan extension in million-molecule challenge. He additionally pointed out the current limitation of aging clocks since they could vary over short time frames and the biological age metrics couldn’t match up with the rate of aging metrics. While AI is the emerging field that is often discussed together with aging, there needs to be a moonshot approach to data acquisition and analysis.

Prof. Morten Scheibye-Knudsen, an associate professor at the University of Copenhagen, presented his work using computational approaches to investigate phenotypes in human aging using datamining of PubMed allowing the identification of diseases of accelerated aging [19, 20]. In extension of this, phenotypes can also be extracted from health care records such as reports from pathologists. His groups analyzed more than 30 million reports identifying patterns of aging at the organismal and tissue level and allowing the identification of longevity compounds by cross-referencing in PubMed. He further described how phenotypes can be extracted from cellular changes at the tissue level, in particular how cellular senescence can be identified using computational investigations of nuclear morphologies [21]. This leads to spatial and single cell senescence scoring that can be used to predict future cancer risk [22, 23]. Importantly, this nuclear senescence predictor can be used in any context where the nucleus of cells can be visualized such as in sputum samples from COPD patients [24] or in blood smears from high-altitude Ethiopian dwellers [25].

Dr. Andrei Tarkhov from Retro Biosciences presented an exciting work on using AI to assist protein design [26]. Their team focused on the Yamanaka factors (OSKM) used in epigenetic reprogramming for reversing aging phenotypes. The wild-type factors are known to perform worse in the cells sourced from old donors. The team attempted to improve the reprogramming efficiency of these factors by directly modifying their amino acid sequences. To make larger edits feasible, they constrained the search space to evolutionarily plausible sequences by training a protein language model, GPT-4b micro, which could be steered by including co-evolving and interacting sequences in its prompt. They guided the model to generate SOX2 and KLF4 variants, modifying up to ⅔ of the wild-type sequences. The re-engineered SOX2 and KLF4 variants improved over wild-type factors by at least two orders of magnitude on day 10 of reprogramming in human dermal fibroblasts. Their results illustrate the promise of AI-assisted protein design for overcoming critical bottlenecks in cellular reprogramming, and in rational engineering of human healthy life extension.

## Stochasticity and dynamics of biomarkers and aging clocks

Focusing on the temporal dynamics of aging biomarkers, this session explored how stochastic fluctuations, emergent system behaviour, and model uncertainty influence the construction, interpretation, and reliability of aging clocks across scales.

In a comprehensive and conceptually rich talk, Prof Andrew Teschendorff from the Shanghai Institute for Nutrition and Health presented advances in the modeling and interpretation of epigenetic clocks, focusing on the distinction between stochastic and non-stochastic components of aging [27]. Using both bulk and single-cell DNA methylation datasets, the team demonstrated that age-associated DNAm changes that are stochastic at the single-cell level, when averaged over millions of cells, enables reliable chronological age prediction, an insight aligned with the central limit theorem [28]. Conversely, many reported instances of biological age acceleration (e.g., smoking or Sars-CoV2 infection) were shown to be largely driven by non-stochastic processes, e.g. cell type composition shifts between immune cells, with one exception being the increased mitotic rate of precancer and cancer lesions. He also introduced cell type-specific clocks for hepatocytes, neurons and glia [29], revealing more robust accelerated epigenetic aging in a range of different liver diseases, as well as a strong acceleration in the glia of the temporal lobe of Alzheimer’s disease patients. Finally, leveraging deep graph neural networks on over a million immune cells, his team identified a ribosomal gene module as a conserved transcriptomic aging signature across tissues and species [30]. The talk concluded by highlighting critical challenges in the field, including immune cell heterogeneity, lack of standardized uncertainty metrics, and the need for single-cell resolution to properly quantify biological aging processes.

Next, Prof. Steffen Rulands from the Ludwig-Maximilians-Universität München emphasized the multi-scale nature of aging - spanning twelve orders of magnitude in time, from molecular binding events to the time scale of organismal aging. Using tools from non-equilibrium statistical physics, including renormalization group theory and field theory, the speaker showed how aging phenomena on long time scales emerge from fast molecular binding events of enzymes with the DNA and chromatin [31]. This leads to a hierarchical model of aging, where different emergent phenomena manifest on distinct temporal scales. The speaker then showed that DNA methylation aging is not only encoded in the longitudinal evolution of DNA methylation marks, but also in how their genomic correlations evolve over time [32]. Through bifurcation analysis and correlation modeling across genomic CpG sites, the team revealed structured, collective behavior —coordinated changes affecting many sites simultaneously— during aging, particularly at the borders of CpG islands, which are influenced by competitive enzymatic activity (TET vs. DNMT3). This interplay was modeled as a phase separation phenomenon, drawing analogies to wetting dynamics in physics. The talk ended with a call to define aging physically—not as a loss of function, but as a slow-timescale dynamic process unfolding across organismal levels—and emphasized that the combination of theoretical biophysics and experimental data can illuminate core mechanisms underlying age-related epigenetic change.

In a talk grounded in statistical physics and systems biology, Prof. Andrew Rutenberg from Dalhousie University presented a phenomenological framework for modeling the dynamics of aging using simple, interpretable equations derived from longitudinal biomarker data. Collaborating with clinicians and gerontologists, the team analyzed standard blood test variables across several cohorts (including the English Longitudinal Study of Aging (ELSA) and kidney failure patients), treating aging as a stochastic, homeostatic process characterized by weak interactions and slow changes. By modeling variables as interacting components within a linear dynamical system, they identified “natural variables” (eigenmodes) with different stability profiles. Crucially, variables that were slow to return to homeostasis and showed age-dependent drift were associated with higher mortality risk—described as “mallostasis”, the failure of stable regulation [33]. The team also observed that noise in the system—representing unmeasured or extrinsic effects—was dynamically coherent, suggesting the existence of latent physiological modules. This approach offers a data-driven lens into the interacting dynamics of biomarkers and may inform how different aging clocks relate and influence one another across time.

In a thought-provoking session on uncertainty in biological age prediction, Dr. Dmitrii Kriukov from the Artificial Intelligence Research Institute in Moscow emphasized that estimating biological age from biomarkers is fundamentally limited by various forms of uncertainty [34]. Since biological age is not a directly measurable quantity, its prediction relies on statistical inference using aging clocks – models trained on biomarkers such as DNA methylation, gene expression, or blood markers. The speaker highlighted that these models are prone to aleatoric (data noise), epistemic (knowledge gap), and model (design) uncertainty [35], especially when applied to out-of-domain samples like reprogrammed or cancer cells. Using examples such as reprogramming-induced rejuvenation and cancer epigenetics, the talk showed that clocks may falsely indicate “youthfulness” in biologically unrelated states. To address this, the speaker proposed three key strategies: check for dataset shifts, use uncertainty-aware models, and critically assess whether predictions are valid given the model’s training domain. The session concluded with a call for developing more robust, mechanistically grounded tools beyond aging clocks to reliably detect true rejuvenation.

## Lifelong dynamics: insights into developmental processes and aging patterns

Spanning early embryogenesis to late-life functional decline, this session and discussion was chaired by Prof. Koh from the National University of Singapore and explored the lifelong dynamics of aging, revealing how damage accumulation, epigenetic drift, reproductive aging, and organ-specific trajectories contribute to heterogeneous aging patterns.

Prof. Vadim Gladyshev from the Harvard Medical School introduced insights into aging, longevity, and rejuvenation, presenting aging as fundamentally driven by the accumulation of molecular damage [36] *–* termed the deleteriome *–* reflecting entropy and disorder across all biological levels [37]. He underscored that although damage unavoidably accumulates [37], it can be diluted through cellular division [38]. His study on metabolite diversity [39] in fruit flies revealed that aging is marked by a progressive increase in metabolic complexity, and his dietary studies demonstrated that organisms fed with nutrients derived from younger sources exhibit extended lifespans [40], thereby providing experimental evidence for the causal role of molecular damage in aging. Additionally, Prof. Gladyshev’s single-cell epigenetic research showed that DNA methylation changes with age can be categorized into co-regulated, mixed, and predominantly stochastic alterations [41], reinforcing the view that aging is largely the result of accumulation of damage at multiple levels. Using an elegant metaphor of a river flowing from mountain to ocean under gravity, he illustrated that aging is like gravity *–* an inevitable force that defines damage accumulation *–* while the duration of this flow symbolizes longevity, which can be modulated by geroprotective interventions akin to building dams. He further introduced a rejuvenation model based on embryogenesis, where biological age decreases from the zygote to a lowest point “ground zero” before increasing toward birth, suggesting that aging processes begin in early embryogenesis, around the stage of gastrulation [42, 43]. He briefly presented his work on heterochronic parabiosis and other stress-induced aging studies, indicating that biological age can reversibly fluctuate due to severe stresses like surgery or COVID-19, demonstrating its reversibility upon recovery [44]. Finally, he emphasized that aging is organ- and system-specific, with different tissues aging at distinct rates [45], reinforcing that while aging is physically inevitable and manifests chemically, it can be mitigated biologically through targeted interventions.

Prof. Nir Eynon (Monash University) outlined his lab’s work on the Multi-Tissue Human Atlas Project, which aims to identify age-related epigenetic marks across diverse tissues and uncover molecular targets that mitigate aging via exercise interventions. He highlighted the roles of differentially methylated positions (DMPs), which reflect predictable age-associated change, and variably methylated positions (VMPs), which capture individual-specific epigenetic variability. His team’s findings on age-related DNA methylation showed that 47.9% of the blood methylome is differentially methylated and 35.2% is variably methylated, with a 59% overlap between these two classes of CpG (methylation) site [46]. These shifts contribute to increased methylation entropy, as many DMPs approach 50% methylation with age, indicating a loss of epigenetic stability over time. He then shared unpublished results comparing tissue-specific methylation changes, showing that brain methylation remains relatively stable, while adipose and skeletal muscle display greater variability and age-related shifts. He emphasized skeletal muscle’s critical role in aging, noting that despite its natural decline, exercise can improve muscle health even later in life [47]. He also introduced the Muscle Epigenetic Age Test (MEAT) clock, developed in collaboration with Steve Horvath, as the first-generation epigenetic clock specifically designed for human skeletal muscle. Trained on muscle methylation data, the MEAT clock accurately predicts chronological age, although it does not necessarily reflect biological aging or functional decline [48]. Finally, he presented evidence that higher aerobic fitness (measured by VO_2_ max) is linked to a younger muscle methylome and transcriptome [49], and that even just four weeks of exercise intervention can shift DNA methylation toward a younger state, while muscle disuse accelerates transcriptomic aging, underscoring exercise as a key modulator of skeletal muscle aging.

Dr. Haiyang Wang, from the Mechanobiology Institute at the National University of Singapore started by emphasizing the importance of female reproductive longevity given that 1 in 6 people globally are infertile and the low total fertility rate in many Asian countries. Key reasons for decreased age-related fertility include reduced egg quantity and egg quality, which can be caused by genomic aneuploidies, mitochondrial dysfunction and poor egg maturation. His talk focused on solutions to the latter problem. During his work at the Mechanobiology Institute in Singapore, Dr. Wang found that the Arp2/3 protein complex is necessary for correct spindle positioning after fertilization. Using ex vivo maturation approaches he later showed that an aged egg surrounded by young granulosa cells (a younger follicular environment) leads to oocyte rejuvenation [50, 51], including improved spindle alignment, mitochondrial membrane potential and reduced aneuploidy, ultimately resulting in more live births to mothers that have undergone such rejuvenating IVF. These improvements appear to be mediated via TZP gap junctions and the transfer of young factors. Future work aims to extend these findings to human cells.

In his short talk Dr. Leong Kim Whye, also from the Mechanobiology Institute in Singapore, continued the theme of female reproductive longevity. Combining detailed experimental quantification and computational modelling, he provided a new biophysical framework for understanding lumen formation during antral follicle development in murine ovaries. His results suggest that follicular fluid formation is governed by a regime transition from near-critical interstitial gaps to phase-separated large lumen. His insight into the underlying mechanism of lumen formation in ovarian follicles may inspire novel interventions for functional and healthy maturation of antral follicles, ultimately addressing the reproductive challenges associated with their age-related reduction in volume and quality [52].

## Complex systems and connectivity: a network approach to aging and metabolism

Framing aging as a problem of systemic connectivity and failure propagation, this session showcased how network science, percolation models, and entropy analysis can quantify system robustness, predict collapse thresholds, and guide the design of targeted geroprotective strategies.

Prof. Ee Hou Yong from the Nanyang University of Technology presented how network science can model aging as a process of damage accumulation across interconnected biological systems. He explained that failures in highly connected nodes (“hubs”) can trigger cascading breakdowns, while random damage causes network fragmentation only beyond a critical threshold [53]. Because scale-free networks are especially vulnerable to hub disruption, resilience can be improved by reinforcing links among peripheral nodes [54]. Using computational models, Prof. Yong showed that system vitality declines gradually before collapsing rapidly at a percolation threshold, mirroring late-life morbidity patterns [55]. He described “bang-bang” repair strategies - all-or-nothing policies in which cells either repair damage at maximum intensity or not at all - that optimize the balance between repair costs and healthspan benefits, and evolutionary simulations in which low-fitness nodes are replaced by new ones to model both short-term and long-term adaptations [56, 57]. He noted that linear networks can be more easily controlled, whereas nonlinear aging dynamics require optimized targeting of key nodes [58]. Because energy costs scale with the extent of control, interventions should prioritize the most critical nodes to maximize healthspan [59]. Prof. Yong concluded that aging is nonlinear and tissue-specific, and that network science offers a valuable framework for understanding and designing effective longevity interventions.

Prof. Feng Ling (Complex Systems Group, IHPC, A*STAR; NUS Physics) advanced percolation theory to model connectivity transitions in complex networks, such as viral outbreaks evolving into pandemics and viral information spreading over online social networks. His work models various forms of percolation mechanisms over networks, from single layer to interdependent networks that exhibit abrupt phase transitions due to cascading failures, exemplified by social media platforms [60]. His influencer node identification algorithm outperformed traditional methods by targeting nodes critical to systemic connectivity of the network [61]. He reviewed some applications extended to neuroscience by others, where percolation thresholds serve as biomarkers for neurodegeneration—studies in fruit flies and Alzheimer’s mouse models revealed declining network robustness [62, 63]. Prof. Feng’s research underscores percolation theory’s versatility in analyzing network resilience, from social systems to biological aging, by quantifying how local disruptions trigger global collapse.

Weihan Huai from Dr. Brian Kennedy’s lab at the National University of Singapore began by addressing the fundamental question in geroscience: Can aging be cured? His research focuses on identifying geroprotectors that can delay or reverse aspects of the aging process. Many geroprotectors, such as metformin and rapamycin, are already used for other medical purposes, making them easier to study and apply in aging research. These compounds have shown promise in extending lifespan in model organisms like *C. elegans*, *Drosophila*, and mice [64, 65]. Huai developed a network-based methodology to identify geroprotectors by mapping aging-associated transcriptomic changes. They employed two strategies: the mimic approach, which replicates gene expression signatures of known longevity-extending compounds, and the reversal approach, which aims to counteract age-related transcriptional changes. Using a *C. elegans* PCA-based transcriptomic clock, they observed that while most lifespan-extending drugs made worms appear transcriptomically “older,” rapamycin showed unique rejuvenation effects. This highlighted the need to distinguish between damage-causing and adaptive transcriptional changes in aging networks. To address this, Huai modelled the aging transcriptome as an interactome comprising aging modules and longevity modules. Their approach involved constructing networks, identifying longevity-associated modules, and screening compounds that suppress aging modules while activating longevity modules. This strategy successfully enriched known geroprotectors and identified novel candidates. Screening top-predicted compounds revealed that many significantly extended nematode lifespans, demonstrating the predictive power of this network-based strategy. Future work aims to refine module definitions and expand validation to mammalian models.

Dr. Csaba Kerepesi from the HUN-REN SZTAKI in Budapest presented novel approaches to quantify biological aging through entropy measurements in DNA methylation patterns and molecular networks. His work establishes entropy - the measure of disorder in biological systems - as a fundamental biomarker of aging. Studies demonstrate that DNA methylation entropy increases with age across mammals. Methylation entropy may be used as an alternative measurement of aging. Notably, the demographically non-aging naked mole-rats show increasing methylation entropy [66], while high-capacity runner rats exhibit reduced organ-specific methylation entropy [67]. Dr. Kerepesi showed results on age-related network entropy changes. His team analysed dMRI-based brain graphs from subjects aged between 42 and 95 years (available at https://braingraph.org/) and found a surprising decrease in (Shannon entropy-based) network entropy with age. They also analysed single-cell protein-protein interaction networks inferred from the single-cell blood transcriptomes of subjects of different ages. They again observed a slight decrease in network entropy with age in adults, and a network entropy increase during early embryonic development. In summary, these preliminary, and still unpublished results, show that network entropy decreases with age in adults and increases during rejuvenation, which is the opposite of what we would expect from the results of regular non-network-based entropy measurements. To find a good interpretation of these results requires more research.

## Quantitative insights into metabolism and longevity

Focusing on metabolism as a measurable and modifiable driver of longevity, the last session showcased how multi-organ metabolomics, lipid network robustness, ribosomal remodeling, and biomarker-guided interventions are shaping predictive and translational strategies in aging research.

Dr. Peter James Mullen from the University of Southern California presented a comparative biology approach to identify metabolic drivers of aging across species, sexes, and organs. His work integrates LC-MS/MS based metabolomics and lipidomics to map age-related metabolic changes in mice, humans, rhesus macaques, killifish, axolotls, and rats [68]. Key findings reveal organ-specific metabolic dysregulation during aging, with thymic involution linked to decreased nucleotide levels and reduced proliferation markers in older mice. Notably, female mice exhibit higher thymic nucleotide levels than males, suggesting sex-dependent metabolic aging patterns. Dr. Mullen’s team developed metabolic clocks that predict biological age using metabolite abundance, identifying known candidates like alpha-ketoglutarate, which extends lifespan in model organisms and inhibits cancer cell growth. These clocks highlight metabolic pathways as potential targets for gerotherapeutics. Multi-organ analyses demonstrate distinct age-related shifts in lipid and nucleotide metabolism, with thymus, liver, and brain showing divergent trajectories. For example, decreased nucleotides in aging thymus correlate with immune decline, while lipidomic changes in other organs reflect systemic metabolic remodeling. Current work focuses on translating these findings into interventions that modulate metabolic pathways to delay aging. The metabolic atlas generated by Dr. Mullen’s lab provides a roadmap for targeting organ- and sex-specific drivers of aging, offering strategies to mitigate age-related diseases and enhance healthspan.

Dr. Max Unfried, a Research Fellow at the National University of Singapore presented a network-based approach to comparative lipidomics, investigating how lipid metabolism contributes to lifespan variation across species. His research reveals that lipids which are critical for membrane structure, signaling, energy storage, and transport, contain molecular signatures capable of predicting maximum lifespan. Key findings demonstrate that principal lipid components (PCs) correlate strongly with maximum lifespan across species. Membrane-associated lipids like phosphatidylcholines (PC/PC-O) and phosphatidylethanolamines (PE/PE-P) show lifespan-related patterns, alongside triglycerides (energy metabolism) and sphingolipids (inflammaging and stress response). Network analyses reveal longer-lived species exhibit more robust lipid interaction networks, with higher edge density, average node degree, and clustering coefficients—suggesting evolutionary optimization of lipidome stability. Dr. Unfried also introduced LipidClock [69], a predictive model using lipidomic data to estimate biological age. Training a LipidClock on pathological samples of pancreatic cancer patients, this clock identifies ceramides, sphingomyelins, and glycerophosphocholines as key mortality-linked lipids [70], and can also predict biological age of healthy individuals. The research bridges lipidomics with evolutionary biology, showing how lipid network robustness may underpin species-specific longevity. Future directions include refining lipid-based biomarkers for aging interventions and disease prognosis.

Dr. Yumi Kim from the biotech company ieumBio investigates how aging alters the structure and function of ribosomes in skeletal muscle, using the African turquoise killifish (*Nothobranchius furzeri*), a short-lived vertebrate model with conserved aging traits [71]. Her research focused on age-related shifts in ribosomal composition and function [72]. Building on emerging evidence that ribosomal heterogeneity confers specialized regulatory capacity yet remains largely uncharacterized in aging, Dr. Kim identifies age-dependent differences in ribosomal protein (RP) regulation at both the mRNA and protein levels, along with variations in RP association with ribosomes and RP-mediated rRNA protection. Using high-resolution cryo-EM, Dr. Kim further demonstrates distinct structural alterations between young and aged ribosomes. These structural and compositional changes correspond to an increased recruitment of ribosome-associated quality-control factors, including Ltn1 and components of the proteasome complex, suggesting that age-associated alterations in ribosomal structure and composition play a critical role in shaping protein homeostasis. This remodeling is closely associated with declines in muscle integrity and reduced locomotor performance in *N. furzeri*. Collectively, Dr. Kim advances a broader understanding of how core elements of the translational machinery are dynamically reconfigured during aging and how such remodeling shapes muscle maintenance and proteostasis.

Finally, Prof. Brian Kennedy from the National University of Singapore addressed critical gaps in aging research, focusing on the interplay between interventions, biomarkers, and clinical translation. His work explores whether interventions like alpha-ketoglutarate (AKG) normalize or extend longevity, comparing strategies in mice and humans [73]. Key questions include whether geroprotectors merely normalize aging processes in humans, similar to calorie restriction in mice, and whether maximum lifespan is modifiable in humans [74]. Prof. Kennedy also highlighted challenges with biomarkers, including reproducibility issues and limited overlap between different aging clocks [75]. He emphasized the need for actionable biomarkers that assess biological age, predict disease progression, and guide clinical interventions. His framework aims to bridge research and clinical practice by developing clinical clocks that identify healthy aging signatures and targets for intervention [76].

## Short talks

An array of short-format presentations was part of the event, providing concise overviews of ongoing projects and offering a snapshot of current innovation, methodological refinement, and conceptual reassessment in the aging field.

Dr. Weilan Wang (National University of Singapore) discussed the translational potential of drug repurposing for gerotherapeutics, emphasizing lower costs, faster timelines, higher approval rates, and opportunities for combination and personalized approaches [77]. Building on a systematic prioritization of FDA-approved candidates scored by preclinical and clinical evidence [78], she shared unpublished data from the RESORT cohort — a longitudinal study of older adults in geriatric rehabilitation. Metformin, ACE inhibitors/ARBs, and aspirin were each independently associated with reduced all-cause mortality. Patients receiving ≥3 such drugs showed greater survival benefits than those on 0–2, hinting at additive/synergistic effects. Wang argued these results offer early clinical support for “poly-gerotherapy” using safe, multi-target drugs to bolster resilience in late life.

Dr. Glen Pridham from Dalhousie University presented a stochastic dynamical modeling approach to population-level aging health trajectories in terms of changes in robustness and resilience, drawing conceptually from Clegg et al. [79]. He modelled longitudinal transition rates in 30+ binary health variables (healthy = 0; deficit = 1) that included signs, symptoms, disabilities and chronic diseases. Damage event rates (0**→**1) were used to characterize robustness, and repair event rates (1**→**0) were used to characterize resilience. Both robustness and resilience were modelled as functions of chronological age and frailty index. The analysis used two large scale longitudinal health studies (HRS and ELSA) and revealed a critical tipping point near age 75, where repair capacity becomes insufficient to offset damage accumulation, leading to runaway frailty index. The dynamical model output was visualized as a velocity field, illustrating the direction and magnitude of health change across age and frailty, with a sharp inflection in the nullcline (df/dt = 0) delineating the onset of irreversible health decline at advanced age [80]. Pridham concluded by highlighting the need to uncover biological mechanisms underlying this systemic collapse, noting preliminary evidence that proteomic, epigenetic, and cardiometabolic biomarkers exhibit parallel inflections near this age.

Kamil Pabis (National University of Singapore) presented a critical re-evaluation of mouse longevity research, demonstrating that apparently impressive lifespan extensions frequently arise from artefactually short-lived control cohorts and regression to the mean [81]. He proposed the “900-day rule” — requiring median control lifespans of approximately 900 days (~30 months) under optimal husbandry — as a pragmatic threshold to distinguish genuine lifespan extension from mere normalization from an unhealthy baseline. Reanalysis of published datasets revealed that only a small minority of interventions retain significant effects above this benchmark. Pabis advocated enhanced husbandry standards, use of historical controls, and methodological rigour to ensure robust, reproducible progress in geroscience.

### One minute poster pitch session

A highlight of the conference was a dynamic one-minute poster pitch session, in which 49 emerging scientists presented concise snapshots of their research, capturing the remarkable breadth of contemporary approaches in aging biology. The overarching topics ranged from molecular and cellular mechanisms and neurobiological models to advanced computational methods, regenerative therapies, and population-level studies. Presenters explored critical aspects such as epigenetic clock benchmarking, modulation of AMPK deficiency, translational control for proteostasis enhancement, neurodegenerative disease modeling, circadian disruptions, and AI-driven strategies for drug discovery and biomarker identification. Broader evolutionary and societal dimensions were also addressed, including cross-species genomic analyses, dietary interventions, and public health insights into frailty and chronic diseases, highlighting the field’s increasingly interdisciplinary nature and its translational potential.

## Panel on the future of Gerophysics

The conference concluded with a panel discussion on he Future of Gerophysics, moderated by Dr. Sebastien Thuault and featuring Prof. Uri Alon, Dr. Peter Fedichev, Prof. Marija Cvijovic, and Prof. Jan Gruber. The panelists emphasized the need for simplicity in describing aging’s complexity, advocating for universal frameworks that bridge thermodynamics (entropy, temperature) and network theory. A central debate revolved around whether new computational or experimental tools are essential, with consensus emerging that iterative collaboration—where models guide experiments and data refines models—is more critical than novel tools alone. The discussion highlighted how physics-inspired “toy models” can reveal aging’s fundamental properties, while machine learning deciphers high-dimensional biomarker data, offering a hybrid path to precision health [82, 83].

A recurring theme was the need to balance mechanistic detail with phenomenological clarity. While microscopic models illuminate pathways and targets, they can become unwieldy when stretched across scales. Phenomenological, coarse-grained theories such as the minimal model [10] and the saturated removal model [1] can help to distill aging into a small set of variables that explain regimes across species, including the striking case of negligible senescence. These models provide quantitative laws linking resilience, entropy, and survival, and in doing so, suggest a path toward more meaningful interventions in humans.

The panellists noted that interdisciplinary communication remains a hurdle, urging joint training initiatives to align the languages of biology, physics, and computational science. Despite aging’s inherent heterogeneity, the session closed on a note of optimism: converging methodologies, from network theory to generative AI, are poised to redefine healthspan interventions. The principal conclusion was that progress in this field will be driven by the synergy of diverse approaches, where “physics predicts, biology validates, and computation unifies”.

## CONCLUSION AND FUTURE OUTLOOK

The field of Gerophysics is getting traction with dedicated workshops [83], and this inaugural conference, attracting more physicists to aging research, and aging researchers being open minded to physics-based approaches. Future initiatives could include summer schools to train the next generation in interdisciplinary approaches. The conference underscored the transformative potential of Gerophysics in unifying aging science through physical principles.

The consensus was that the future of Gerophysics will not lie in one paradigm alone, but in the integration of mechanistic, AI-driven, and phenomenological approaches, each informing the other. This synthesis will enable Gerophysics to mature into a discipline that is predictive, testable, and ultimately able to guide therapies that extend healthy human lifespan.

To sustain momentum, we propose 1.) annual conferences alternating between Asia, Europe, and the Americas, 2.) dedicated funding for collaborative grants in Gerophysics, 3.) online repositories for shared models, datasets, and benchmarks.

By 2030, these efforts could establish Gerophysics as a cornerstone of geroscience.

The conundrum of aging is that it is highly complex, with species, tissue and individual differences impacting the progressive decline that occurs over time; yet single gene or drug interventions can delay the process. Given the long timeline of aging, even in most vertebrate animal models, and the fact that almost everything changes with age, molecular biology has been of limited value. Biological approaches have clearly defined molecular pathways that contribute to age and produced targets for intervention; however, they have not yet succeeded in integrating these pathways into an explainable model of aging. Much of the motivation for establishing Gerophysics as a field is that it will not be truly possible to understand aging unless it is reduced to physical principles and perhaps accompanying mathematical models. At the core, aging is a process of network decline and theoretical physics-based strategies may unravel its complexity and reduce it to an explainable phenomenon under a predictive framework, uniting biology and physics into testable laws.
